# The After-Care Association

**Published:** 1903-03-14

**Authors:** 


### THE AFTER-CARE ASSOCIATION.

Sir William Church presided at the annual meeting of the After-Care Association for poor persons discharged recovered from asylums, and pointed out that the Association assisted those who were prepared and willing to help themselves. The State made provision for all those mentally-afflicted persons whose family and friends were unable to maintain them, but it considered that when they recovered they should be able to maintain themselves. This was, however, a most difficult thing for these poor people to do for several reasons—for one thing, the world looked with suspicion on all those who were, or had been, mentally afflicted, and they were consequently ostracised by all their former acquaintances. Then, again, although recovered from their malady they were usually quite unfit to take up any regular work just at first, especially if it were of a trying character, and if compelled to do so frequently became victims once more of their old disorder. The After-Care Association stepped in at this juncture and obtained for them an interval of change of scene. The Association in the course of last year had assisted 221 such cases. Every case was thoroughly investigated. The boarding out of cases in cottage homes in the country had proved a great success.

